# Nature's weapons: Bioactive compounds as anti-cancer agents

**DOI:** 10.3934/publichealth.2024038

**Published:** 2024-06-18

**Authors:** Amogh Auti, Madhura Tathode, Maria Michela Marino, Antonio Vitiello, Andrea Ballini, Francesco Miele, Valeria Mazzone, Alessia Ambrosino, Mariarosaria Boccellino

**Affiliations:** 1 Department of Precision Medicine, Università della Campania “Luigi Vanvitelli”, 80138 Naples, Italy; 2 Ministry of Health, Directorate-General for Health Prevention, 00144 Rome, Italy; 3 Department of Clinical and Experimental Medicine, University of Foggia, Via Rovelli 50, 71122, Foggia, Italy; 4 General Surgery Unit, Università degli Studi della Campania “Luigi Vanvitelli”, 80138 Naples, Italy; 5 Department of Experimental Medicine, Università della Campania “Luigi Vanvitelli”, 80138 Naples, Italy; 6 Department of Life Sciences, Health and Health Professions, Link Campus University, 00165 Rome, Italy

**Keywords:** phytochemicals, chemopreventive agents, anti-inflammatory compounds, neuroprotective agents, antioxidant compounds

## Abstract

Cancer represents a major global health burden, prompting continuous research for effective therapeutic strategies. Natural compounds derived from plants have emerged as potential strategies for preventing cancer and treatment because of their inherent pharmacological properties. This comprehensive review aimed to evaluate the therapeutic potential of five key natural compounds: apigenin, quercetin, piperine, curcumin, and resveratrol in cancer prevention and therapy. By examining their molecular mechanisms and preclinical evidence, this review seeks to elucidate their role as potential adjuvants or stand-alone therapies in cancer management. The exploration of natural compounds as cancer therapeutics offers several advantages, including low toxicity, wide availability, and compatibility with conventional chemotherapeutic agents. We highlighted the current understanding of their anticancer mechanisms and clinical applications for advancing personalized cancer care to improve patient outcomes. We discussed the empirical findings from in vitro, in vivo, and clinical studies reporting biological activity and therapeutic efficacy in antioxidant, immunomodulatory, anti-carcinogenic, and chemo-sensitizing modes. Innovative delivery systems and personalized treatment approaches may further enhance their bioavailability and therapeutic utility in a synergistic approach with chemo- and radiotherapeutic disease management. This review underscores the importance of natural compounds in cancer prevention and treatment, promoting a multidisciplinary approach to the development of innovative therapeutic strategies.

## 1. Introduction

Ayurveda is a 5000-year-old healing science originating in India with the concept of application of natural ingredients for health purposes such as curcumin, quercetin, piperine, resveratrol, etc. Most of the Ayurvedic bioactive compounds (ABCs) are phytochemicals derived from different parts of various types of herbs, shrubs, and plants. ABCs can potentially be used to control chronic health problems such as obesity through the targeted and controlled release of bioactive compounds [1]–[3]. All of these natural compounds can be categorized into different chemical types. These include phenols, terpenoids, thiols, dietary fiber, polyphenols, flavonoids, anthocyanidins, phenolic acids, curcuminoids, alkaloids, carbohydrates, carotenoids, vitamins, phytosterols, polyunsaturated lipids, and organosulfur compounds [4]. These compounds have diverse pharmacological properties, including antioxidant, anti-inflammatory, antibacterial, immunomodulatory, anticancer, anti-proliferative, antimutagenic, and antithrombotic activities, and possess great application potential for practice in nutraceuticals and pharmaceutical therapeutics [5]–[8]. The use of these ABCs in complex foods is limited because they are sensitive to various factors such as pH and temperature. Hence, comprehensive research of identification and characterization of these compounds can be challenging due to their chemical diversity and the need for extraction and separation techniques [9]. The drug discovery process has seen phytochemicals used to discover new insights about their therapeutic utility. Flavonoids, a class of polyphenolic compounds, are significant plant-derived chemicals containing a benzopyrone moiety, and they have been demonstrated to affect various biological processes.

Carcinogenesis or oncogenesis is a multistep process where genetic and environmental factors are required to come together for cellular transformation and overpassing the apoptosis. The first stage of carcinogenesis starts with converging multiple irreversible genetic mutations causing deregulation of cell cycle control. This promotes selective growth of the initial cells and their progeny by enhancing mitogenic signaling. Finaly, uncontrolled cellular proliferations begin to spread and invade (locally or distantly) the tissue resulting in bad to worst outcomes. Inflammatory response is a natural process against injury, infection, and tumor cells by the immune system of the body. Cytokines and chemokines play crucial roles as regulators of inflammatory signaling in tumor promotion. Hence, clinical trials investigating anti-cytokine and anti-chemokine receptor therapies have shown promising effects [10],[11]. Current research trends in cancer therapy focus on the surge of immune cell infiltration in the tumor microenvironment (TME) and their interaction with stroma and tumor cells. Cancer-associated inflammation has chronological aspects that can be explained in three ways, i.e., autoimmunity, cancer therapy-induced inflammation, and tumor-induced inflammation. Single cell transcriptomics revealed tumor-associated fibroblasts and immune cells increase pro-tumorigenic inflammation. The dynamics of tumor immunology resulted in promising therapy developments in the invading anti-tumor immunological checkpoint blockade by novel immunotherapies like cancer vaccines, bi-specific anti-PDL1 antibodies, and T cells engineered with chimeric antigen receptors (CARs) [12].

As mentioned above, the master regulator NF-κB-signaling axis mediated proinflammatory factors IL-1α, IL-1β, IL-6, TNF-α, CXCL-1, and CXCL-2 promote cell proliferation through STAT3 mediated activation of the MCL-1 oncogene [10],[13]. The TNF cytokine family members TNFα, TNF-β, and IL-1 cytokines in TME constitutively activate transcription factors NF-κB, AP1 (JUN/FOS), and STAT3 through respective transmembrane receptors to support oncogenic pathways [14],[15]. Hence, NF-κB initiated proinflammatory signaling has a pivotal function in the onset and advancement of cancer through immune dysregulation and oncogene expression in cancer stem cells [16]. This inflammatory feedback loop exerts to drug resistance in breast cancer as well as establishes autocrine and paracrine regulation of mitogen in TME [17],[18]. Transforming growth factor β (TGF-β) is secreted by tumor cells and considered as a multipotent cytokine that influences TME by inducing vascularization, strong immunosuppression in T and NK cells, and tumor progression to mesenchymal transition via SMAD pathway activation [19]–[21].

## 2. What are chemopreventive agents and how do they work?

Chemopreventive agents are substances that aid in the prevention or progression of cancer cells whereas chemotherapeutic agents are used after confirmatory cancer diagnosis [22]. These agents can be natural or synthetic and work by various mechanisms, such as protecting DNA from ROS-induced damage, inhibiting cell proliferation, and promoting apoptosis in cancer cells. Chemopreventive agents are classified in three types based on the effective stage of action in carcinogenesis to progression. The compounds classified as primary can inhibit the genotoxic effects of carcinogens at the pre-cancerous stage. The secondary prevention strategy blocks the development of a malignant phenotype. Preventive cancer diagnoses, such as screening through mammography in the high-risk female population or through colonoscopy for colorectal cancers, are considered as risk-reducing secondary preventive strategies [23]. The tertiary agents prevent the recurrence of cancer after successful therapeutic or surgical management of tumors [24],[25]. Temoxifen, a selective estrogen receptor modulator, was the first drug approved by the FDA to be used to reduce ER-positive breast cancer among the high-risk population [26]–[28]. Metformin [29], RhoA/Rho kinase, and Ras/ERK pathway inhibitor statin drugs [30]–[32], as well as aspirin, a selective COX-2 inhibitor classified as a nonsteroidal anti-inflammatory drug (NSAID) [33], are widely used in clinical practice. Additionally, IL-1 cytokine inhibitors [34],[35] are also employed for their action in delaying or preventing carcinogenesis. BV and HPV viral vaccines are best examples of immunoprevention of the transformation by viral infection. These preventive vaccines were introduced in national immunization programs of developed countries [36]–[38].

Phytochemicals, naturally occurring compounds found in plants, have gained attention as potential chemopreventive agents. They exhibit anti-cancer properties by modulating inflammatory, hormonal, cell division, and redox homeostasis signaling pathways in turn inducing apoptosis, and inhibiting tumor growth [39]. However, chemical chemotherapeutic agents, while effective in treating cancer, can have limitations and side effects, including toxicity to normal cells, development of drug resistance, and adverse effects on the immune system. Phytochemicals have demonstrated potential for anti-inflammatory effects by regulating the canonical pathways involved in inflammation [40]. Many such active compounds are known to possess protective properties against side effects of cancer therapies if in combination with chemotherapeutic drugs. Isothiocyanates and curcumin are highlighted as natural phytochemicals with significant anti-inflammatory and cancer chemopreventive effects [41]. Scientists have been focusing on improving therapy results by applying precision medicine and specifically targeting tumors with personalized medicine, combining chemotherapeutic side effect alleviating drugs and natural compounds [42],[43].

## 3. Phytochemicals and cancer

Several ABCs have been shown to possess potential anti-cancer effects by regulating molecular pathways that are implicated in the growth and progression of cancer. These mechanisms include increasing antioxidant potential, inhibiting proliferation, inducing cell cycle arrest and apoptosis, and regulating the immune system [44],[45]. Any plausible drug candidate is studied for its physicochemical and pharmacokinetic characteristics. In this review, we discuss the biochemical propensity and various biological properties of active phytochemicals such as curcumin, resveratrol, apigenin, quercetin, and piperine.

**Figure 1.**
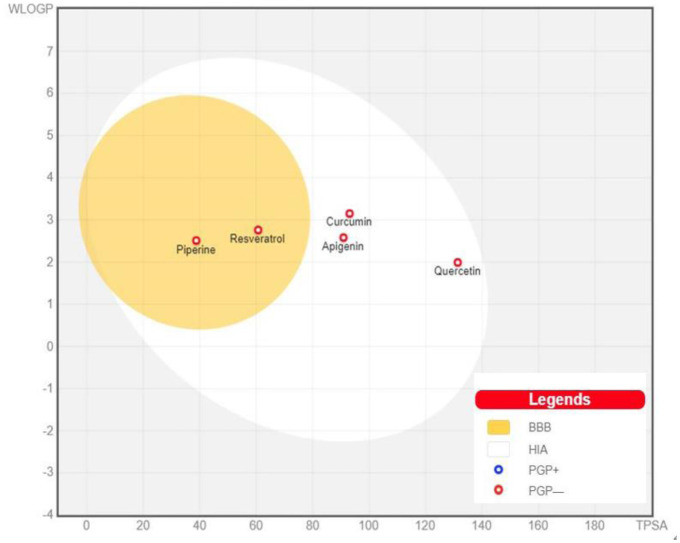
The estimations for passive human gastrointestinal absorption (HIA) and blood-brain barrier (BBB) permeation are both illustrated through the BOILED-Egg plot. This predictive model is centered on determining whether a small molecule compound is likely to function as a substrate or inhibitor of proteins that influence critical pharmacokinetic characteristics. Permeability glycoprotein (P-gp) indicates that substrate molecules actively efflux through biological membranes. All of the bioactive compounds in this review article are non-P-gp substrates. Piperine and resveratrol are predicted candidates for BBB permeation.

The graph generated on SwissADME (Figure 1) allows the probabilistic assessment of passive gastrointestinal absorption (HIA) and brain permeability (BBB) in relation to the positioning of the molecules or active phytochemical compounds concerning the water partition coefficient (WLOGP) and topological polar surface area (TPSA). Molecules within the white area are predicted to have a high probability of passive absorption through the gastrointestinal tract, while those within the yellow region (yolk) are expected to have a high likelihood of crossing the blood-brain barrier. The white and yellow regions do not exclude each other, meaning that all of the molecules in the yolk region are equally permissible through HIA but not inversely. Additionally, the blue points indicate molecules predicted to be actively effluxed by P-gp (PGP+), while the red points signify those predicted as non-substrates of P-gp (PGP−) [46],[47]. The five substances discussed in this review are PGP-ve suggesting their intracellular accumulation. The blood-brain barrier consists of a monolayer of endothelial cells associated with astrocytes and pericytes. This lining forms a selective filtering barrier that prevents the passage of large molecules, heavy metals, drug compounds, and pesticides to protect the nervous system from toxic and infectious agents [48]. BBB remains a major obstacle to treat neurological degenerative, as well as proliferative, diseases due to the selective passage and lack of proinflammatory activity.

## 4. Some phytochemicals

### 4.1. Curcumin

Curcumin [1,7-bis(4-hydroxy-3-methoxyphenyl)-1,6-heptadiene-3,5-dione] is a lipophilic flavonoid compound found in the rhizome of *Curcuma longa*, commonly known as turmeric. Chemically, curcumin is composed of flanking two oxy-substituted aryl groups on seven carbon chain. This group participates in biological redox reaction giving antioxidant capacity to the stressed cell. In *in vivo* experiments, curcumin showed potent antioxidant [49], anti-inflammatory [50],[51], neuroprotective [52],[53], and antitumor [54],[55] properties by activating nuclear factor erythroid 2-related factor 2 (Nrf2) driven pathways. Poor bioavailability of curcumin limits its potential therapeutic use in clinical settings. However, studies on various curcumin derivatives, analogues, nano-carriers, and combination formulas are gaining more attention to improve bioavailability of curcumin [56]. Curcumin and its analogues possess pathway modulating potential by binding it to various cellular proteins, receptors, and channels either through covalent, non-covalent hydrophilic, or hydrogen bonding. Studies reveal TNF-α, Cox-1, Cox-2, Bcl-2 [57], HDAC [58], DNMT-1, and HIV-1 integrase protein [59] are some binding partners of curcumin. Compounds like resveratrol, piperine, catechins, quercetin, and genistein in combination have also been reported to be synergistic with curcumin action of alleviation of inflammation. The therapeutic effects of curcumin are found to be blunted because of i) low water solubility, and ii) faster metabolism and elimination from the system [60]. A pharmacokinetic study reports the serum bioavailability of curcumin in rats and humans was enhanced by 125% and 2000% when administered in combination with piperine. Piperine enhances intestinal absorption, serum concentration, and bio availability of curcumin [61]. Studies report that, despite having poor BBB crossing properties, this does not limit its therapeutic effects on neuronal diseases. Curcumin present in essential oils has been demonstrated to penetrate the BBB and exert a protective effect against aluminum chloride (AlCl_3_)-induced neurotoxicity in the brains of Swiss albino mice. The turmeric extract in essential oils improved curcumin bioavailability by more than 10 folds in comparison to curcumin without essential oils [62]. The formulation of curcumin derivatives dispersed in the phospholipid matrix when given to 19 patients with Parkinson's disease. Patients in the treatment arm showed detectable curcumin in cerebrospinal fluid (CSF) and reduced phosphorylated α-synuclein (p-syn) load in neurons of a skin biopsy. Moreover, the curcuminoid-treated group showed a decrease of COMPASS-31 and NMSS scores, and improved clinical parameters, than untreated patients [63].

Recent studies emphasize the significance of Nrf2 (nuclear factor-erythroid 2-related factor 2) as a crucial transcription factor in defending against inflammation, oxidative, and electrophilic stresses. Research with diverse animal models has shown that Nrf2 deficiency heightens susceptibility to various cancers, suggesting its role as a tumor suppressor in preclinical models. Conversely, hyperactivated Nrf2 has been linked to several tumors and unfavorable prognoses [64],[65]. The zinc-curcumin compound, Zn(II)-curc, has demonstrated the ability to activate Nrf2, exhibiting anticancer effects in various cancer cell lines. This compound increases levels of heme oxygenase-1 (HO-1), p62/SQSTM1, and Nrf2 while decreasing the Nrf2 inhibitor Keap1 [66]. The interaction between p62/SQSTM1 and Nrf2 may increase treatment effectiveness. Additionally, the anti-inflammatory and antioxidative characteristics of curcumin are linked to its modulation of the Nrf2 pathway, which enhances antioxidant pathways and glutathione production while improving insulin resistance.

Cisplatin is a primary chemotherapeutic drug used against many cancers due to its cytotoxic action of inhibiting DNA transcription and replication in actively proliferating cells. However, it confers off-target effects as multi-organ toxicity makes this drug less useful in cancer treatment. The combination therapy with natural compounds approach has given new hope to minimize the side effects and improve the efficacy of chemotherapeutic drugs [67],[68]. The co-treatment of cisplatin with curcumin induced apoptosis by activating NRF2 and inhibited ovarian cancer plasticity marker endothelin-1 (ET-1) in SKOV3 cells as well as in a rat model. The study showed curcumin treatment improved the expression of PGC-1α and TFAM (mitochondrial biogenesis markers) and prevented renal fibrosis [69]. Patients with high NCL and PD-L1 expressing TNBC showed faster cancer progression and poor outcomes. In an in vitro study on NCL^High^ /PD-L1^High^ MDA-MB-231 and NCL^High^/PD-L1^High^ HCC70 TNBC cells treated with curcumin resulted downregulation of PD-L1 expression and effective cytotoxic activity by NCL-specific T cells.

Curcumin has been found to stimulate intracellular autophagy [70]. Curcumin is renowned for its anti-inflammatory attributes shown by inhibiting the proinflammatory cytokine activated JAK/STAT3 pathway. In a study involving tumor-associated fibroblasts (TAF) from gastric cancer in vitro, co-culturing and mouse xenograft induced the chemo-resistant gastric cancer phenotype. Curcumin treatment abrogated the chemoresistance by strongly suppressing IL-6 and IL-8 expression in co-cultured CAF by suppressing the JAK/STAT3 pathway [71].

A clinical trial, employing a randomized, double-blind, placebo-controlled design, was conducted to investigate the potential of curcumin in mitigating severe dermatitis induced by radiation therapy among 30 breast cancer patients. The curcumin treatment alleviated severe radiation-induced dermatitis at the end of the trial compared to the placebo [72]. Tumor necrosis factor-α (TNF-α) is a pivotal inflammatory mediator in TME and is on the radar of many anti-inflammatory as well as anti-cancer treatments. A meta-analysis published on the eight randomized control trail on patients subjected to curcumin supplementation as intervention showed significant reduction in circulating TNF-α [73]. In a separate randomized controlled trial involving patients with stage IIB-IIIB cervical carcinoma, curcumin intervention before radiation therapy induced radio-sensitization and better therapy outcome by reducing serum survivin protein levels in 75% of patients. Whereas, 60% of patients found elevated serum survivin protein in the placebo arm [74]. The current ongoing clinical trials are described in Table 1.

### 4.2. Apigenin

Apigenin is derived naturally from a variety of fruits, vegetables, and Chinese medicinal herbs. It has been extensively studied for its potential anti-cancer effects and low toxicity. Studies have indicated that apigenin exhibits suppressive effects on various human cancers both in laboratory settings and in living organisms, achieving this through a range of biological mechanisms. These include the initiation of cell apoptosis and autophagy, induction of cell cycle arrest, inhibition of cell migration and invasion, and promotion of an immune response [75],[76]. Studies have reported that apigenin modulates several cell signaling pathways, including the PI3K/AKT, MAPK/ERK, JAK/STAT, NF-κB, and Wnt/β-catenin pathways. Moreover, apigenin has been extensively researched for its potential in cancer prevention and treatment by influencing various cellular processes, such as tumor suppression, angiogenesis, apoptosis, cell cycle regulation, and inflammation, as well as by targeting PI3K/AKT, NF-κB, MAPK/ERK, and STAT3 pathways [77].

Epidemiological research highlights that the introduction of natural substances into the diet can slow down the development of tumors, especially female tumors such as cervical, ovarian, and breast cancer. Zhang et al. conducted research investigating the anti-tumor impact of apigenin on breast cancer, examining its effects both in laboratory settings using 4T1 cells and in living organisms on a xenografted tumor mouse model. The study revealed that apigenin triggers apoptosis in breast cancer cells through modulation of the PI3K/AKT/Nrf2 pathway. Additionally, apigenin has been shown to enhance the immune microenvironment of tumors in mice afflicted with breast tumors, ultimately suppressing breast cancer growth. As a result, apigenin emerges as a hopeful option for breast cancer treatment [78]. Alternative splicing (AS) plays a crucial role in the diversification of cancer-specific transcriptomes. Triple-negative breast cancer (TNBC) is known for its resilience against cancer-specific transcriptome modifications. Aberrant splicing isoforms of the TNBC transcriptome promote tumor development and resistance. In this context, the identification of strategies to reprogram AS circuits in the direction of transcriptomes that, in addition to improving the response to therapy, delay tumor growth, becomes fundamental. Apigenin, linked with splicing factors like heterogeneous nuclear ribonucleoprotein A2 (hnRNPA2), has the ability to reshape the AS transcriptome associated with TNBC. Notably, apigenin-mediated AS events were found to be significantly enriched in substrates of hnRNPA2. Through comparative transcriptomic analyzes of human TNBC tumors and non-tumorous tissues, it was highlighted that apigenin contributes to shifting the alternative splicing isoforms (ASI) associated with cancer toward non-tumorous ones, with a preference for anti-apoptotic and cell proliferation factors. In vivo, in TNBC xenografted mice, apigenin altered cancer-associated aberrant ASI, reducing cell proliferation and increasing pro-apoptotic ASI [79].

It is known that conventional anti-tumor therapies, in most cases, lead to adverse effects and are sometimes not effective. Often the anticancer properties of natural substances, such as apigenin, are slowed down by their low solubility in water. For this reason, nanostructured lipid carriers (NLCs) encapsulating apigenin (APG-NLC) with a lipid matrix containing rosehip oil, a known anti-inflammatory and antioxidant, have been developed. APG-NLC was optimized, achieving an average particle size of less than 200 nm, a surface charge of −20 mV, and an encapsulation efficiency of more than 99%. In vitro studies demonstrated significant antiangiogenic activity of APG-NLC in ovo and selective antiproliferative effects on various tumor cell lines, without inducing toxicity in healthy cells [80]. Yang et al. utilized a copolymer commonly used in drug delivery, Poly (lactic-co-glycolic acid) (PLGA), along with hyaluronic acid (HA) to prepare HA-coated PLGA nanoparticles that are specific for CD44 receptors on colon cancer cells with high CD44 expression. Apigenin was encapsulated in PLGA to create PLGA-API-NPs, which were subsequently coated first with chitosan and then with HA, resulting in HA-PLGA-API-NPs which showed a stronger and more prolonged release ability. These nanoparticles have demonstrated efficacy as a drug delivery system for API in treating colon tumors with high CD44 expression. Specifically, in nude mice, HA-PLGA-API-NPs exhibited enhanced targeting precision for the HT-29 ectopic tumor model compared to PLGA-API-NPs [81].

Apigenin is able to inhibit hypoxia-inducible factor (HIF)-1α expression in tumor cells exposed to hypoxic conditions. Abnormal myocardial glucolipid metabolism is often associated with an increase in HIF-1α in hypertension-induced cardiac hypertrophy. In rats with renovascular hypertension-induced cardiac hypertrophy, oral administration of 50–100 mg/kg of apigenin for 4 weeks led to decreased myocardial HIF-1α protein expression. This treatment also resulted in reductions in blood pressure, heart weight, heart weight index, cardiomyocyte cross-sectional area, as well as serum angiotensin II and serum and myocardial free fatty acids. Moreover, the administration of apigenin enhanced the expression of myocardial peroxisome proliferator-activated receptor (PPAR) α, carnitine palmitoyltransferase (CPT)-1, and pyruvate dehydrogenase kinase (PDK)-4 proteins. Furthermore, it attenuated the expression of myocardial PPARγ, glycerol-3-phosphate acyltransferase genes (GPAT), and glucose transporter (GLUT)-4 proteins. The suppression of HIF-1α expression and the upregulation of PPARα and its downstream genes CPT-1 and PDK-4 may represent the molecular mechanisms driving these effects [82]. In prostate cancer, mortality is primarily linked to the emergence of metastases rather than the primary, localized disease [83]. Apigenin hampers the onset of prostate carcinogenesis by altering TGF-β-activated pathways linked with cancer advancement and metastasis, notably the Smad2/3 and Src/FAK/Akt pathways in prostate tissue. Vascular endothelial growth factor (VEGF) plays a pivotal role as an angiogenesis regulator; transforming growth factor-β1 (TGF-β1) induces VEGF expression in human prostate cancer PC3-M and LNCaP C4-2B cells. Apigenin treatment markedly decreases VEGF production. Additionally, apigenin impedes TGF-β1-triggered phosphorylation and nuclear translocation of Smad2 and Smad3. Targeted transient suppression of Smad2 or Smad3 attenuates apigenin's impact on VEGF expression. Apigenin also hinders Src, FAK, and Akt phosphorylation in these cells. The inhibitory effect of apigenin on VEGF expression and Smad2/3 phosphorylation is reversed by constitutively active Src [84]. Apigenin has shown antiproliferative and antiangiogenic effects in pancreatic cancer, increasing its potential as a chemopreventive agent. The expression of GLUT-1 was markedly elevated in pancreatic adenocarcinoma specimens compared to adjacent controls. Under hypoxic conditions, the expression of HIF-1α, GLUT-1, and VEGF proteins was induced in CD18 and S2-013 pancreatic cancer cells. In vitro experiments carried out in the same cells under both hypoxic and normoxic conditions demonstrated that apigenin suppressed the mRNA and protein expression of HIF-1α, GLUT-1, and VEGF. Additionally, apigenin impeded the hypoxia-induced upregulation of GLUT-1 and VEGF mRNA in both cell lines [85].

### 4.3. Quercetin

Quercetin (QC), a flavonoid found in a variety of fruits and vegetables, has garnered attention for its antioxidant, anti-inflammatory, and anti-cancer characteristics. As a potent scavenger of ROS, QC shields cells from oxidative harm. It has been extensively investigated for its potential in cancer prevention and treatment, attributed to its capacity to impede cancer cell proliferation and trigger apoptosis. QC has demonstrated encouraging outcomes in suppressing the proliferation of colorectal, breast, prostate, and lung cancer cells. Additionally, it exerts anti-cancer effects by inhibiting DNA damage, reducing inflammation, and modulating cell signaling pathways. QC-loaded hyaluronic acid-modified nanoliposomes (LP-Quer-HA) were employed to target prostate cancer stem cells (CSCs) that overexpress CD44+ receptors. The use of these nanoliposomes as a QC delivery system increased its potency at lower concentrations, effectively diminishing the CD44+ cell population and preventing the proliferation and migration of prostate cancer cells. In particular, administration of 10 µM free QC reduced the viability of androgen-resistant PC3 cells by 16%; the utilization of nanoliposomes, LP-Quer-HA, loaded with the same concentration significantly increased cell death by up to 60%. This effect was associated with upregulation of cytochrome c, Bax, and caspases 3 and 8, and the downregulation of survivin and Bcl-2 expression. Moreover, LP-Quer-HA upregulated the expression of E-cadherin and reduced the expression of fibronectin, N-cadherin, and MMP9, inhibiting cell migration and invasion. In PC3 cell tumor spheroids treated with LP-Quer-HA, a decrease in the number of CD44 cells and a reduction in the expression of CD44, Oct3/4, and Wnt were observed. Furthermore, LP-Quer-HA inhibited p-ERK expression while increasing p38/MAPK and NF-κB protein expression. In androgen-sensitive LNCaP cells, LP-Quer-HA demonstrated considerable efficacy, reducing cell viability by 10% to 52% compared to free QC [86].

Furthermore, a reduction in necrosis, fibrosis, and anti-programmed cell death 1 (PD-L1) expression in liver tissues was observed. Wu et al. investigated the impact and underlying mechanism of a combined treatment approach utilizing QC and the anti-PD-1 antibody on hepatocellular carcinoma. This combination therapy enhanced macrophage immunity and increased the expression of CD8a, CD4, CD11b, interleukin (IL)-10, and interferon (IFN)-γ, while decreasing the expression of IL-4, IL-6, toll-like receptor 4 (TLR4), inhibitor of nuclear factor κBα (IκBα), and the p65 subunit of NFκB. Additionally, a decrease in necrotic and fibrotic tissue, as well as a decrease in PD-L1 expression in liver tissues, was observed. Besides, it positively influenced gut microbiota (GM) diversity, favoring specific bacterial groups. Numerous studies have indicated a potential link between microbiota and inflammation, as well as various diseases, including those affecting gastrointestinal health, immune responses, neurological conditions, and cancer [87]–[90]. The upregulation of M2 macrophage-associated genes, including arginase-1 (Arg-1), IL-10, transforming growth factor-β (TGF-β), and matrix metalloproteinase-9 (MMP-9), was increased, while genes associated with M1 macrophages, including IL-6, IL-12a, IL-1β, and tumor necrosis factor-α (TNF-α), were decreased. Furthermore, the combined therapy alleviated gut microbiota dysbiosis and enhanced the abundance of *Firmicutes*, *Actinobacteria*, and *Verrucomicrobiota* at the phylum level, as well as *Dubosiella* and *Akkermansia* at the genus level [91].

QC exerts a regulatory effect on ER stress-mediated apoptosis. In an in vivo Wistar albino rat model of experimental inflammatory bowel disease (IBD) induced by trinitrobenzene sulfonic acid (TNBS), elevated disease activity levels and indices of oxidative stress, inflammation markers, along with increased immunoreactivities of NF-κB and c-Jun N-terminal mitogen-activated protein kinase were observed in the colons of the TNBS colitis group. Additionally, increased immunoreactivity of glucose regulatory protein 78 and caspase-12, as well as epithelial cell apoptosis, were demonstrated in the colon. However, QC administration in the TNBS+QC group ameliorated the induced histopathological alterations, apoptosis, inflammation, oxidative stress, and ER stress [92].

QC has the potential to improve airway barrier function and alleviate barrier impairment induced by inflammatory factors. QC treatment increased transepithelial electrical resistance and reduced substance leakage across cell layers in a Calu-3 airway epithelial cell culture model. QC also induced changes in the composition of tight junctional proteins and partially inhibited cell replication, leading to a decrease in linear junctional density. All of these alterations led to an improvement in barrier function. In addition, QC was also effective in mitigating barrier impairment induced by the proinflammatory cytokine TNF-α by reducing the increase in ERK 1/2 caused by TNF-α [93]. To achieve targeted delivery of QC as a therapeutic agent to HepG2 tumor cells, QC was encapsulated within a synthesized nanocarrier (NC) made up of Fe_2_O_3_/starch/polyvinyl alcohol (Fe_2_O_3_/S/PVA NC). In the NC containing Fe_2_O_3_ nanoparticles, the drug loading percentage (DLE) and encapsulation efficiency (EE) of QC reached 47% and 86.50%, respectively, while in the NC without Fe_2_O_3_, they were 36% and 73%, respectively. The release pattern of QC in acidic and natural media demonstrated controlled release properties that were pH-dependent, characteristic of the nanocarrier. Viability assessments performed on L929 and HepG2 cells treated with Fe_2_O_3_/S/PVA/QC, using MTT staining and flow cytometry, underscored the efficacy of the nanocarrier against HepG2 tumor cells [94].

Quercetin (QC) can alleviate hypoxic-ischemic brain damage (HIBD)-induced neurodegeneration by regulating autophagy and NLRX1 expression. 36 seven-day-old Sprague-Dawley rats were divided into control, QC, HI, and HI + QC groups. HIBD was induced using the Rice method in HI and HI + QC rats, subjected to 2 hours of hypoxia (8% oxygen) after ligation of the left common carotid artery. The HI + QC group received intraperitoneal injection of QC (30 mg/kg) 1 hour before hypoxia, while the QC group received only QC. HI + QC groups showed significantly improved escape latencies and platform crossing times. Additionally, there was a noteworthy reduction in infarct volume, along with an increase in the number of autophagic bodies and a decrease in apoptotic cells. Transmission electron microscopy revealed improved brain tissue morphologies, and immunofluorescence staining showed upregulated expressions of NLRX1, ATG7, and Beclin1, coupled with downregulated expressions of mTOR and TIM23, LC3B protein level, and LC3II/LC3I ratio [95]. QC improves pulmonary hypertension in rats exposed to chronic hypoxic conditions by promoting apoptosis through the HMGB1/RAGE/NF-κB pathway. In rats subjected to daily hypoxia (8-10 hours) for 4 weeks, with intragastric administration of 100 mg/kg of QC before each hypoxic exposure resulted in a significant reduction in right ventricular hypertrophy (RVHI) and right ventricular systolic pressure (RVSP). Furthermore, QC pretreatment mitigated pulmonary vascular remodeling and improved right heart function compared to rats exposed to normoxia. In addition, QC influenced protein expressions in lung tissue, reducing levels of HMGB1, RAGE, NF-κB, and Bcl-2, while increasing Bax and cleaved caspase-3 [96].

QC demonstrates protective efficacy in ameliorating kidney dysfunction under hypobaric hypoxia. In rats subjected to simulated hypobaric hypoxia at 7620 m, it was observed that maximum renal damage occurred 12 hours after exposure, as evidenced by an increase in oxidative stress (ROS, MDA), renal metabolites (creatinine, urea nitrogen in the blood and uric acid), and by a reduction in antioxidants (GSH) in the plasma. QC administration 1 hour before hypoxia exposure reduced oxidative stress, creatinine, and blood urea nitrogen levels, stabilized HIF-1α protein expression, reduced VEGF protein expression, and reduced LDH levels in the kidneys. The optimal dose of QC administered 1 hour before exposure to hypoxia was 50 mg/kg body weight. Histopathological observations confirmed the protective effects of QC in preventing hypobaric hypoxia-induced renal damage by reducing oxidative stress in rats [97].

### 4.4. Piperine

Piperine is a pungent alkaloid and bioactive compound found in black pepper and certain Piper species. Black pepper is used in many Indian traditional medicinal compositions in the treatment of respiratory and digestive tract disorders to enhance the bioavailability of several other natural compounds [98]. Piperidine is a N-acylpiperidine (CH2)_5_NH substituted by a (1E,3E)-1-(1,3-benzodioxol-5-yl)-5-oxopenta-1,3-dien-5-yl group at the nitrogen atom for piperine. Piperine has been found to enhance the antioxidant system, increase the levels and activity of detoxifying enzymes, and suppress stem cell self-renewal, contributing to its chemopreventive effects. Piperine exhibits potent anti-inflammatory properties across various models, as demonstrated by numerous studies [99]. In vitro experiments reveal its ability to inhibit inflammatory factors and oxidative stress-associated genes in different cell types, such as nucleus pulposus cells and human peripheral blood mononuclear cells (PBMCs). Piperine also effectively reduces ROS/RNS production and downregulates the expression of inflammatory mediators like p38, JNK, AP-1, iNOS, and COX-2. Moreover, it inhibits the production of pro-inflammatory cytokines and prostaglandins in microglial cells and down-regulates pathways associated with inflammation and NF-κB activation. In vivo studies support these findings, demonstrating a dose-dependent reduction in inflammation across various experimental models, including paw edema, arthritis, and granuloma formation. Additionally, piperine demonstrates efficacy in reducing inflammatory markers and symptoms in conditions like rheumatoid arthritis. Overall, the consistent and robust anti-inflammatory effects of piperine underscore its potential for investigating inflammatory-related conditions in future studies [99].

Anna Greenshields and colleagues established that, in vivo, a TNBC (by MDA-MB-231 and MDA-MB-468 cells) tumor when treated with piperine selectively inhibited tumor growth and progression to EMT with no significant adverse effect on normal mammary cells. The effect of piperine was evidenced by low G1 and G2 driving gene expression with increased cyclin dependent kinase inhibitor p21^Waf1/Cip1^ expression. The tumor progression marker MMP2 and MMP9 expression was found to be low in the same experiment [100].

Moreover, the compound hinders the proliferation and viability of cancer cell lines by regulating cell cycle progression and exhibiting anti-apoptotic properties. Piperine also influences the activity of enzymes and transcription factors to hinder invasion, metastasis, and angiogenesis. Significantly, piperine demonstrates antimutagenic effects and suppresses the activity and expression of multidrug resistance transporters like P-gp and MRP-1. Crucially, the examined studies consistently indicate the selective cytotoxicity of piperine toward cancer cells in comparison to normal cells.

### 4.5. Resveratrol

Resveratrol (RSV) (3,5,4′-trihydroxystilbene) is a natural polyphenolic phytoalexin found in grapes, berries, and red wine. RSV belongs to the chemical class of stilbenoid, a derivate of stilbene. Its antioxidant properties have been extensively investigated in the context of aging and age-related diseases. RSV activates various antioxidant enzymes, enhances mitochondrial function, and scavenges free radicals. In terms of its anti-cancer potential, resveratrol has demonstrated inhibitory effects on the growth of several cancer cell types, including breast, prostate, liver, and colon cancer. It modulates multiple signaling pathways involved in cell cycle regulation, apoptosis, and inflammation, thereby impeding tumor growth, e.g., inhibiting cervical cancer progression to metastasis by inhibiting STAT3 phosphorylation [101]. RSV has been shown to cross BBB enabling its antioxidant and anti-inflammatory potential effects in neurological diseases [102].

**Figure 2.**
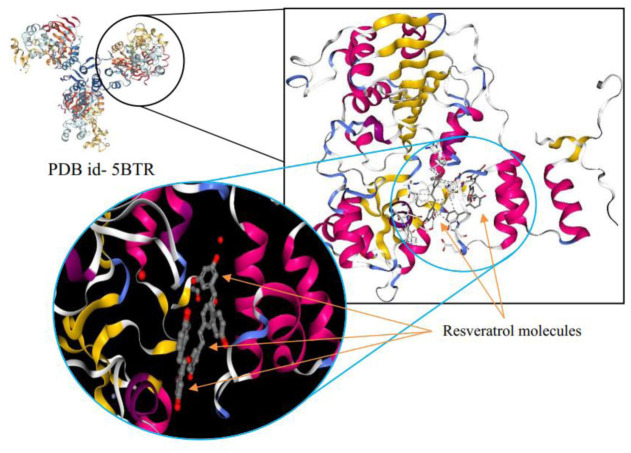
The crystal structure of PDB id-5BTR showing 3 RSV molecules allosterically interacting with the SIRT-1 N-terminal domain.

Sirtuins are NAD-dependent protein deacetylases enzymes, which catalyze the de-acetylation of an acetylated lysine residue with the help of the nicotinamide group from NAD, resulting in a deacetylated protein. 3 RSV molecules activate SIRT1 by allosteric binding and a substrate dependent manner (Figure 2) [103]. When activated, SIRT1 disrupts the TLR4/NF-κB/STAT axis, decreasing cytokine production by inactive immune cells, and suppressing pro-inflammatory factors released by macrophages/mast cells, including the platelet-activating factor and TNF-α [104]. Trauma hemorrhage induces immune cell infiltration along with neutrophils in response to locally produced cytokines and chemokines. Proinflammatory cytokines TNF-α and IL-6 are important chemotactic stimuli for neutrophils, T cells, and NK cells to initiate inflammation. An RSV dose resulted in estrogen receptor-dependent upregulation of the p38 MAPK/heme oxygenase 1 pathway and suppressed the proinflammatory cytokine effects in rats [105].

**Figure 3.**
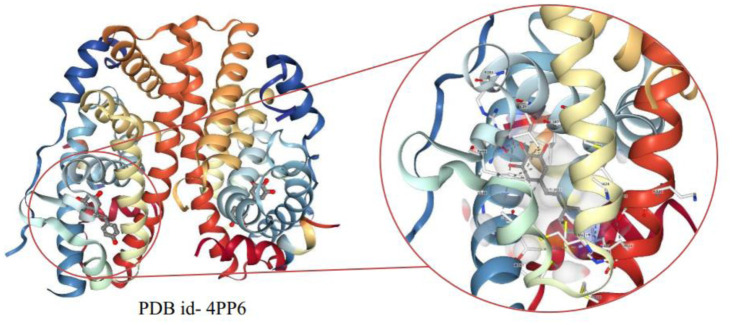
The crystal structure of PDB id- 4PP6 of the estrogen receptor α (ERα) ligand binding domain (LBD) complexed with a molecule of resveratrol.

The cytoplasmic NF-κB is an inactive transcription factor complexed physically with an inhibitor of NF-κB - IκBα. During inflammation or stress, IKKβ- and NEMO-dependent phosphorylation and following the degradation of IκB proteins activates NF-κB. As a result, the separation of IκB proteins from the NF-κB complex exposes the nuclear localization sequence (NLS) of p65 and p50 leading to nuclear translocation and promotes expression of the target proinflammatory genes [106]. Active SIRT2 mediated deacetylation of lysine residues of p65 inhibit its nuclear localization and interaction with the target gene promotor on the chromatin [107]. Estrogen receptor α (ERα) acetylation enhances DNA binding and trans-activation activities of ERα by promoting interaction with cell cycle and apoptosis regulator 1 (CCAR1) coregulator and co-activator. RSV has been found to bind directly (see Figure 3) to ERα resulting in a disruption in co-regulator binding as well as activating SIRT1 to de-acetylate ERα abrogating chromatin binding [108]. RSV treatment suppressed stem cell markers and induced apoptosis in TNF-β stimulated 5-fluorouracil resistant CRC cells. Moreover, resveratrol downregulated inflammatory axis NF-κB, CXCR4, and EMT markers E-cadherin in the same study [109]. Many clinical trials undertake RSV as a dietary supplement or a drug for cancer patients to assess chemopreventive or therapeutic sensitization effects (see Table 1).

Preclinical investigations have demonstrated the efficacy of these phytochemicals against diverse cancer types. Additionally, some of these phytochemicals are currently under clinical trials for testing their efficacies as chemotherapeutic combination drugs against various cancers [110].

## 5. Future opportunities

Leveraging artificial intelligence (AI) drug design can accelerate the discovery of promising natural compounds. AI-powered virtual screening of vast drug libraries of chemically modified natural compounds can identify candidates with optimal therapeutic potential [111]. Synthetic biology and metabolic engineering of pathways to synthesize specifically modified compounds with desirable properties can lead to reliable solutions for therapeutic purposes. The successful synthesis of nanocurcumin, for example, demonstrates the potential of this approach to improved bioavailability and biological retention. Furthermore, advancements in bioinformatics, particularly high-throughput genomic and proteomic data analysis combined with omics integration strategies, can shed light on mechanisms by which these phytochemicals cure disease at the single-cell level. Novel targeted therapies utilizing biocompatible carriers or packaging nanoparticles can deliver these compounds directly to the TME. This formulation strategy can enhance targeting specificity and biological activity by intratumoral drug accumulation.

**Table 1.** Phase 2 and phase 3 clinical trial studies on curcumin, piperine, resveratrol, and quercetin on cancer patients with the NIH clinical research trial number (NCT) listed on ClinicalTrials.gov.

**NCT No.**	**Title**	**Type of Cancer**	**Interventions**	**Phase**

	**Curcumin**
NCT03769766	Clinical trial of curcumin for preventing the progression of low-risk prostate cancer under active surveillance	Prostate Cancer	Drug: Curcumin and Placebo	Phase 3
NCT00295035	Phase III trial of gemcitabine, curcumin, and Celebrex in patients with metastatic colon cancer	Colon Neoplasm	Drug: CELECOXIB Drug: CURCUMIN	Phase 3
NCT02064673	Assessment of recurrence-free survival in patients who underwent radical prostatectomy with adjuvant curcumin	Prostate Cancer	Drug: Curcumin Drug: placebo	Phase 3
NCT00486460	Phase III trial of gemcitabine, curcumin, and Celebrex in patients with advanced or inoperable pancreatic cancer	Pancreatic Cancer	Drug: Gemcitabine Drug: Curcumin Drug: Celebrex	Phase 3
NCT01246973	Oral curcumin for radiation dermatitis	Radiation-induced Dermatitis	Drug: Curcumin Drug: Placebo	Phase 2 and Phase 3
	**Curcumin + Piperine**
NCT06080841	Curcumin supplementation in cervical cancer	Locally Advanced Cervical Cancer	Dietary Supplement: Curcumin Dietary Supplement: Curcumin + Piperine	NA
NCT04731844	Curcumin and piperine in patients on surveillance for monoclonal gammopathy, smoldering myeloma, or prostate cancer	Prostate Cancer, Multiple Myeloma, Smoldering Multiple Myeloma (SMM)	Drug: Curcumin plus Piperine	Phase 2
NCT06063486	Curcumin to improve inflammation and symptoms in patients with clonal cytopenia of undetermined significance, low risk myelodysplastic syndrome, and myeloproliferative neoplasms	Clonal Cytopenia of Undetermined Significance, Essential Thrombocythemia, Myelodysplastic Syndrome	Dietary Supplement: Curcumin/Demethoxycurcumin/Bisdemethoxycurcumin-containing supplements with Piperine Extract, Drug: Placebo	Phase 2
	**Resveratrol**
NCT01476592	A biological study investigating Resveratrol's impact on notch-1 signaling in individuals with low-grade gastrointestinal tumors	Neuroendocrine Tumor	Dietary Supplement Resveratrol	NA
NCT03482401	Distribution of dietary polyphenols and methylxanthines in the breast tissue of breast cancer patients	Breast Cancer	Dietary Supplement Polyphenol	NA
NCT05306002	Nutritional intervention and DNA damage of patients with HBOC	HBOC Syndrome	Antioxidant therapy	NA
NCT03253913	Resveratrol and Sirolimus in lymphangioleiomyomatosis trial	Lymphangioleiomyomatosis	Drug Sirolimus Drug: Resveratrol	Phase 2
NCT04867252	Effects of combined Resveratrol and Myo-inositol on altered metabolic, endocrine parameters, and perceived stress in patients with polycystic ovarian syndrome	PCOS	Drug: Resveratrol, Myoinositol, Drug: Metformin, Pioglitazone	Phase 2
	**Quercetin**
NCT01538316	Prostate cancer prevention trial with Quercetin and Genistein	Primary Prevention of Prostate Cancer	Dietary Supplement: Quercetin, Genistein Supplement: Placebo	Not Applicable
NCT04733534	An open-label intervention trial to mitigate senescence and enhance resilience in adult survivors of pediatric cancer	Childhood Cancer	Drug: Dasatinib plus Quercetin, Drug: Fisetin	Phase 2
NCT05456022	Therapeutic efficacy of quercetin versus its encapsulated nanoparticle on the tongue squamous cell carcinoma cell line	Oral Cancer	Drug: Quercetin-encapsulated PLGA-PEG nanoparticles (Nano-QUT), Drug: Doxorubicin chemotherapy	Phase 2
NCT02195232	Cancer associated thrombosis and isoquercetin (CATIQ)	Thromboembolism of Vein VTE in Colorectal Cancer, in Pancreatic Cancer, and in Non-small Cell Lung Cancer	Drug: Isoquercetin	Phase 2 and Phase 3
NCT03493997	Multicentre international study for the prevention with Ialuril® of radio-induced cystitis (MISTIC)	Prostate Cancer	Combination Product: Radiotherapy + IAluril® + Ialuril Soft Gels® | Radiation: Radiotherapy only	Phase 2
NCT02446795	Isoquercetin as an adjunct therapy in patients with kidney cancer receiving first-line sunitinib	Renal Cell Carcinoma, Kidney Cancer	Drug: Sunitinib | Drug: Isoquercetin | Drug: Placebo	Phase 1 and Phase 2
NCT05724329	Neoadjuvant tislelizumab in combination with dasatinib and quercetin in resectable HNSCC (COIS-01)	Head and Neck Squamous Cell Carcinomas	Drug: Tislelizumab + Dasatinib + Quercetin (neoadjuvant), and Tislelizumab + Dasatinib + Quercetin (adjuvant)	Phase 2
NCT01732393	Effect of quercetin in prevention and treatment of oral mucositis	Chemotherapy Induced Oral Mucositis	Drug: Oral Quercetin Capsules | Drug: Placebo	Phase 1 and Phase 2
NCT03476330	Chemopreventive effects of quercetin on squamous cell carcinoma in individuals with Fanconi anemia	Fanconi Anemia, Squamous Cell Carcinoma	Drug: Quercetin (dietary supplement)	Phase 2

## 6. Conclusion

Empirical data of natural bioactive phytochemicals have shown potential anti-inflammatory and anti-carcinogenic effects. These phytochemicals act via regulating molecular pathways that inhibit carcinogenesis either by sensitizing the cancer cells to chemotherapies or alleviating the major inflammatory pathways like JAK/STAT and NF-κB in the tumor microenvironment. Our literature review highlights and promotes five natural compounds that have proved their health benefits in clinical trials when used as dietary supplements. Their mechanisms of action include antioxidant effects, modulation of cell signaling pathways, induction of apoptosis, and enhancement of chemosensitivity, which collectively contribute to their anti-cancer properties. Quercetin and resveratrol are distinguished by their ability to interfere with cancer cell proliferation and survival, while curcumin's broad-spectrum anti-inflammatory and anti-cancer effects suggest its utility in overcoming drug resistance. Apigenin and piperine, too, have emerged as potent molecules with capabilities to inhibit tumor growth and improve the efficacy of standard chemotherapeutic agents. Despite the promising data from preclinical models, the transition to clinical application requires further investigation. While the therapeutic potential of these compounds is undeniable, their efficacy in actively combating cancer is hampered by two crucial factors: limited bioavailability and poor biological retention. Therefore, it is prudent to recommend ABCs as regular dietary supplementations, bolstering the natural defense as a preventive measure against carcinogenic diseases. The efficacy and safety of these phytochemicals, alone or in combination with conventional therapies, require validation through clinical trials to establish optimal dosages, therapeutic windows, and potential side effects. Moreover, understanding the bioavailability and metabolism of these compounds in the human body remains critical for their development as therapeutic agents. In conclusion, the integration of phytochemicals into cancer therapy could revolutionize treatment standards, offering more personalized, less toxic, and potentially more effective options.

## Use of AI tools declaration

The authors declare they have not used Artificial Intelligence (AI) tools in the creation of this article.
